# Applications of Next-Generation Sequencing Technologies to Diagnostic Virology

**DOI:** 10.3390/ijms12117861

**Published:** 2011-11-14

**Authors:** Luisa Barzon, Enrico Lavezzo, Valentina Militello, Stefano Toppo, Giorgio Palù

**Affiliations:** 1 Department of Histology, Microbiology, and Medical Biotechnologies, University of Padova, I-35121 Padova, Italy; E-Mails: enrico.lavezzo@unipd.it (E.L.); valentina.militello@unipd.it (V.M.); giorgio.palu@unipd.it (G.P.); 2 Department of Biological Chemistry, University of Padova, I-35121 Padova, Italy; E-Mail: stefano.toppo@unipd.it

**Keywords:** next generation sequencing, deep sequencing, virus discovery, metagenomics, virome, virology, quasispecies, molecular diagnosis, human immunodeficiency virus, drug resistance, minority variants

## Abstract

Novel DNA sequencing techniques, referred to as “next-generation” sequencing (NGS), provide high speed and throughput that can produce an enormous volume of sequences with many possible applications in research and diagnostic settings. In this article, we provide an overview of the many applications of NGS in diagnostic virology. NGS techniques have been used for high-throughput whole viral genome sequencing, such as sequencing of new influenza viruses, for detection of viral genome variability and evolution within the host, such as investigation of human immunodeficiency virus and human hepatitis C virus quasispecies, and monitoring of low-abundance antiviral drug-resistance mutations. NGS techniques have been applied to metagenomics-based strategies for the detection of unexpected disease-associated viruses and for the discovery of novel human viruses, including cancer-related viruses. Finally, the human virome in healthy and disease conditions has been described by NGS-based metagenomics.

## 1. Introduction

Novel DNA sequencing techniques, referred to as “next-generation” sequencing (NGS), provide high speed and throughput that can produce an enormous volume of sequences. The most important advantage provided by these platforms is the determination of the sequence data from single DNA fragments of a library that are segregated in chips, avoiding the need for cloning in vectors prior to sequence acquisition.

The first next-generation high-throughput sequencing technology, the 454 FLX pyrosequencing platform (http://www.454.com/), which was developed by 454 Life Sciences and later bought by Roche, became available in 2005. In early 2007, Illumina released the Genome Analyzer (http://www.illumina.com), developed by Solexa GA, and more recently, SOLiD was released by Applied Biosystems (http://www.appliedbiosystems.com). This field is in rapid expansion and novel and improved platforms are continuously being developed and released, like Heliscope by Helicos (http://www.helicosbio.com/), Ion Torrent PGM by Life Technologies (http://www.iontorrent.com/) and a real-time sequencing platform by Pacific Biosciences (http://www.pacificbiosciences.com/).

While the platform developed by Pacific Biosciences, as well as other novel sequencing platforms, are referred as “third-generation” because they sequence processively single large DNA molecules without the need to halt between read steps, 454 pyrosequencing, Illumina GA and SOLiD methods represent the “second generation” systems, able to sequence populations of amplified template-DNA molecules with a typical “wash-and-scan” technique [1]. Given these criteria, Ion Torrent PGM and Heliscope sit between “second-” and “third-generation” technologies, since they do not completely fulfill the features assigned to each category.

These NGS methods have different underlying biochemistries and differ in sequencing protocol (sequencing by synthesis for 454 pyrosequencing, Illumina GA, Ion Torrent PGM and Heliscope, sequencing by ligation for SOLiD), throughput, and for sequence length (Table 1). Thus, the SOLiD system may be more suitable for applications that require a very high throughput of sequences, but not long reads, such as whole genome re-sequencing or RNA-sequencing projects, while both 454 and Illumina provide data suitable for *de novo* assembly and the relative long length of 454 FLX (and its smaller version GS Junior) reads allows deep sequencing of amplicons, with applications in microbial and viral metagenomics and analysis of viral quasispecies, as described in this review. The technical features of NGS methods (reviewed in refs. [2,3]) will not be described in this review, which is focused on the diagnostic applications of NGS in clinical virology.

## 2. Applications of NGS Technologies to Diagnostic Virology

NGS technologies are currently used for whole genome sequencing, investigation of genome diversity, metagenomics, epigenetics, discovery of non-coding RNAs and protein-binding sites, and gene-expression profiling by RNA sequencing (reviewed in refs. [2–6]). Typical applications of NGS methods in microbiology and virology, besides high-throughput whole genome sequencing, are discovery of new microorganisms and viruses by using metagenomic approaches, investigation of microbial communities in the environment and in human body niches in healthy and disease conditions, analysis of viral genome variability within the host (*i.e.*, quasispecies), detection of low-abundance antiviral drug-resistance mutations in patients with human immunodeficiency virus (HIV) infection or viral hepatitis, as outlined in this review article.

### 2.1. Detection of Unknown Viral Pathogens and Discovery of Novel Viruses

The human population is exposed to an increasing burden of infectious diseases caused by the emergence of new previously unrecognized viruses. Climate changes, globalization, settlements near animal and livestock habitats, and the increased number of immunocompromised people probably contribute to the emergence and spread of new infections [7]. In addition, several clinical syndromes are suspected to be of viral etiology, but the causing agent cannot be isolated and recognized by traditional culture and molecular methods. Thus, there is the need to improve methods for the identification of unsuspected viral pathogens or new viruses. Subtractive techniques, such as representational difference analysis or random sequencing of plasmid libraries of nuclease resistant fragments of viral genomes, have led in the past to the discovery of several viruses, including human herpesvirus type 8 [8], human GB virus [9], Torque Teno Virus [10], bocavirus [11], human parvovirus 4 [12], WU polyomavirus [13] and KI polyomavirus [14]. These techniques are poorly sensitive and time-consuming, and thus are unsuitable for large scale analysis. For these purposes, NGS-based methods have been developed. However, traditional cloning and sequencing methods can be relatively simple and sensitive for the discovery of new viruses when used for the analysis of otherwise sterile samples, and may represent an alternative to NGS. One of these methods is termed VIDISCA (Virus Discovery cDNA Amplified Fragment Length Polymorphism Analysis) and may be applied to sterile specimens, such as cell culture supernatants [15]. In this method, samples are ultracentrifuged for viral particle enrichment and treated by DNase and RNase to digest away cellular nucleic acids. Capsid-protected viral nucleic acids are then purified, converted to double stranded DNA, digested with restriction enzymes and ligated to oligonucleotide adaptors, which are used as primer binding sites for comparative PCR [15]. This method was described originally in the context of the discovery of severe acute respiratory syndrome coronavirus (SARS-CoV) in 2004 [16]. Microarray-based diagnostic assays have also been used to characterize previously unknown viruses, such as SARS-CoVs [17], but require information on the genome of the virus or closely related viruses that are under investigation [18].

High throughput NGS techniques represent a powerful tool which can be applied to metagenomics-based strategies for the detection of unknown disease-associated viruses and for the discovery of novel human viruses [19,20]. Compared with microarray-based assays, NGS methods offer the advantage of higher sensitivity and the potential to detect the full spectrum of viruses, including unknown and unexpected viruses.

One of the first applications of NGS for pathogen discovery was the investigation of three patients who died of a febrile illness a few weeks after transplantation of solid organs from a single donor and for whom conventional microbiological and molecular tests, as well as microarray analysis for a wide range of infectious agents, had not been informative [21]. In this study, RNA was purified from blood, cerebrospinal fluid and tissue specimens from transplant recipients and, after digestion with DNase to eliminate human DNA, RNA was reverse-transcribed and amplified with random primers. Amplification products were pooled and sequenced with the use of the 454 pyrosequencing platform. After subtraction of sequences of vertebrates and highly repetitive sequences, contiguous sequences were assembled and compared with motifs represented in databases of microbes, leading to the identification of putative protein sequences which were consistent with an Old World arenavirus. Additional sequence analysis showed that it was a new arenavirus related to lymphocytic choriomeningitis viruses. Further serological and immunohistochemical analyses documented that the virus was transmitted through organ transplantation [21].

A similar strategy, based on unbiased high-throughput sequencing using 454 pyrosequencing for the direct diagnosis of viral infections in clinical specimens, has been used in different diagnostic settings, such as the investigation of patients during seasonal influenza and norovirus outbreaks [22], the identification of an astrovirus as a causative agent for encephalitis in a boy with agammaglobulinemia, after conventional methods had failed to identify an infectious agent [23], and the identification of a hemorrhagic fever-associated arenavirus from South Africa (Lujo virus) [24].

When implemented into virus-discovery methods based on shotgun sequencing, next-generation technologies greatly enhance turnaround time and sensitivity. For example, the 454 system was implemented into a virus discovery assay based on an improved version of the VIDISCA protocol to minimize rRNA contamination [25]. Likewise, the association of NGS techniques with rolling circle amplification (RCA), another method for virus discovery, could greatly increase its performance. RCA employs the PhiX29 polymerase to selectively amplify small double stranded DNA (dsDNA) molecules and is used to amplify circular genomes of DNA viruses and bacteria plasmids [26]. Recently, RCA led to the identification and whole genome sequencing of novel human papillomaviruses and polyomaviruses [27], including human polyomaviruses 6 and 7 (HPyV6 and HPyV7), detected in cutaneous swab specimens of healthy persons [28], and trichodysplasia spinulosa–associated polyomavirus (TSPyV), detected in skin lesions from immunocompromised patients [29].

Besides 454 pyrosequencing, short-read-based metagenomic methods using the Illumina GA platform have also been used to detect unknown viruses in clinical specimens. The Illumina GA platform allowed to identify influenza A viruses from swab specimens and *de novo* assembly of its genome [30–32]. It also led to the detection of viral pathogens in nasopharyngeal aspirate samples from patients with acute lower respiratory tract infections [33], such as a new enterovirus, named enterovirus 109 (EV109) detected in a cohort of Nicaraguan children with viral respiratory illness [34].

A comparative study of the analytical sensitivity of the two platforms, 454 pyrosequencing and Illumina GA, for the detection of viruses in biological samples was done on a set of samples which were artificially spiked with eleven different viruses [35]. The Illumina method had a much greater sensitivity than 454, approaching that of optimized quantitative real-time PCR. However, at low viral concentration in the specimen, the number of reads generated by the Illumina platform was too small for *de novo* assembly of viral genome sequences [35].

Vector-borne viruses and zoonotic viruses represent another important and challenging field for viral discovery. The feasibility of detecting arthropod-borne viruses was explored in *Aedes aegypti* mosquitoes experimentally infected with dengue virus and pooled with noninfected mosquitoes to simulate samples derived from ongoing arbovirus surveillance programs [36]. Total RNA was purified from mosquito pools, reverse-transcribed using random primers and subjected to 454 pyrosequencing, which led to the correct identification of infected mosquito pools [36].

Another interesting strategy to discover arthropod-borne viruses exploits the property of invertebrates to respond to infection by processing viral RNA genomes into siRNAs of discrete sizes. A recent study on small RNA libraries sequenced by NGS platforms [37] showed that viral small silencing RNAs produced by invertebrate animals are overlapping in sequence and can assemble into long contiguous fragments of the invading viral genome. Based on this finding, an approach of virus discovery in invertebrates by deep sequencing and assembly of total small RNAs was developed and applied to the analysis of contigs (*i.e.*, a contiguous length of genomic sequences in which the order of bases is known to a high confidence level) assembled from published small RNA libraries. Five previously undescribed viruses from cultured Drosophila cells and adult mosquitoes were discovered, including three with a positive-strand RNA genome and two with a dsRNA genome [37]. This strategy for virus discovery based on deep sequencing of small RNAs has been also successfully used in plant virology [38].

Bats are reservoirs for emerging zoonotic viruses that cause diseases in humans and livestock, including lyssaviruses, filoviruses, paramyxoviruses, and SARS-CoV. In a surveillance study focused on the discovery of bat-transmitted pathogens, gastrointestinal tissue obtained from bats was analyzed by coronavirus consensus PCR and unbiased high-throughput pyrosequencing that revealed the presence of sequences of a new coronavirus, related to those of SARS-CoV [39].

### 2.2. Detection of Tumor Viruses

Computational subtraction analysis of data obtained using conventional shotgun sequencing methods has been used to identify viral sequences (e.g., HBV, HCMV, human papillomaviruses 18 and 16, HHV8, HCV, EBV and human spumavirus) in EST libraries derived from normal and cancerous tissues [40] and in post-transplant lymphoproliferative disorder tissue [41]. In these studies, computational subtraction analysis relied on sequence data gathered for other purposes as the yield of viral sequences was very low due to the predominance of human sequences. However, exploiting the great amount of sequencing data achievable by NGS methods, computational subtraction analysis could become a method of choice for viral discovery. This approach has been used for the discovery of a new polyomavirus associated with most cases of Merkel cell carcinoma (MCC) [42]. MCC is a rare and aggressive human skin cancer that typically affects elderly and immunosuppressed individuals, a feature which was suggestive of an infectious origin. RNA was purified from MCC samples and analyzed by 454 pyrosequencing. Digital transcriptome subtraction of all human sequences led to the detection of a fusion transcript between a human receptor tyrosine phosphatase and a Large T antigen sequence related to murine polyomaviruses. This sequence was used as starting point for whole genome sequencing and characterization of this previously unknown polyomavirus that was called Merkel cell polyomavirus (MCPyV). The presence of the virus in 80% MCC tissues but only in about 10% of control tissues from various body sites, including the skin, and the demonstration that, in MCPyV-positive MCCs, viral DNA was integrated within the tumor genome in a clonal pattern, strongly suggested the etiological role of the virus in the pathogenesis of MCC [42].

In a NGS study of the skin virome of a patient with MCC in comparison with healthy controls [43], another human polyomavirus strain was detected, which was nearly identical to the recently discovered HPyV9 polyomavirus [44] and closely related to the lymphotropic polyomavirus (LPV). Likewise, unbiased high-throughput sequencing or deep sequencing of amplicons generated with consensus primers targeting regions of the viral genome conserved within viral families, like the tumor-associated *Polyomaviridae* and *Papillomaviridae*, allowed the discovery and characterization of many new polyomavirus and papillomavirus genotypes in several animal species.

The *Papillomaviridae* family includes several viral species and at least 189 completely characterized papillomavirus types and putative new types are continuously found [45]. High throughput 454 pyrosequencing of amplicons generated by consensus PCR of a conserved region of viral genome was used to detect and genotype HPV in cervical cytology specimens [46]. The method allowed the detection of HPV types which were present in low amount in multiple infections and had the potentiality to detect a broad spectrum of HPV types, subtypes, and variants [46]. A similar approach was used to detect and genotype cutaneous HPV types in a large series of squamous cell carcinoma of the skin and other skin lesions [47]. Several different HPV types were detected, including novel putative cutaneous HPVs [47].

Investigation of retrovirus and retroviral vector integration sites in host cell chromosomes is another field of viral oncology which received a great contribution from NGS technologies. The use of viral vectors that integrate in host genome for gene transfer may cause malignant transformation due to activation of host proto-oncogenes or inactivation of tumor-suppressor genes, as a consequence of viral vector integration within these genes [48–50]. Deep sequencing technology has been used to map the integration sites of retroviruses and HIV [51], as well as retroviral and HIV-based vectors for gene therapy and cell reprogramming [52–54]. Deep sequencing methods for detection of retrovirus integration are based on 454 pyrosequencing of products of ligation-mediated PCR (LM-PCR) [55,56] or linear amplification–mediated PCR (LAM-PCR) [57]. Both LM-PCR and LAM-PCR use restriction enzymes to fragment the DNA of interest containing proviruses. Then, digested DNA is ligated with a compatible linker and amplified by PCR using primers that anneal in the LTR and in the linker sequence. Nested primers containing linkers for the 454 protocol are then used for a second PCR, which is processed by 454 high-throughput sequencing. A LAM-PCR method without the use of restriction enzymes was also developed for high throughput sequencing [58]. Recently, a new method was developed for recovering sites of integrated DNA based on the bacterial transposase MuA. The transposase is used to introduce adaptors into genomic DNA to allow PCR amplification and analysis by 454 pyrosequencing. This method could avoid the bias associated with restriction enzymes and recovered integration sites in a near random fashion. It provided a measure of cell clonal abundance, which is crucial for detecting expansion of cell clones that may be a prelude to malignant transformation [59].

### 2.3. Characterization of the Human Virome

The human microbiome is the entire population of microbes (*i.e.*, bacteria, fungi, and viruses) that colonize the human body. Metagenomics refers to culture-independent studies of the collective set of genomes of mixed microbial communities and applies to explorations of all microbial genomes in consortia that reside in environmental niches, in plants, or in animal hosts, including the human body [60–62]. The “metagenome” of microbial communities that occupy human body niches is estimated to have a gene content approximately 100-fold greater than the human genome [63]. These diverse and complex collections of genes encode a wide array of biochemical and physiological functions that may be relevant in healthy and disease conditions.

Metagenomics strategies are generally based on whole genome shotgun sequencing of nucleic acids purified from a specimen. In case of bacteria metagenomics, analysis can be simplified by exploiting universal and conserved targets, such as 16S rRNA genes, which have both conserved regions that can be targeted by PCR primers, and intervening variable sequences that facilitate genus and species identification [60,61]. At variance, no conserved ubiquitous viral sequences are available for broad amplification of viral genomes and methods to enrich samples with viral particles can only be used. In addition, viral metagenomics analyses, which have been applied so far mostly in environmental samples like fresh water, reused wastewater, and ocean water [64–67], have shown that many of the detected viral sequences are unique and represent unknown viral species. Thus, viral sequences may be missed even by shotgun sequencing [68].

A recent study [69] developed a bioinformatic annotation strategy for identification and quantitative description of human pathogenic viruses in virome data sets and applied this strategy to annotate sequences of viral DNA and RNA (cDNA) extracted from sewage sludge residuals resulting from municipal wastewater treatment (biosolids), which were obtained by 454 pyrosequencing. In this experimental model, within the 51,925 annotated sequences, 94 DNA and 19 RNA sequences were identified as human viruses. Virus diversity included environmentally transmitted agents such as parechovirus, coronavirus, adenovirus and aichi virus, as well as viruses associated with chronic human infections, such as human herpesviruses and hepatitis C virus [69].

In the diagnostic setting, metagenomic approaches could be used for systematic analysis of samples collected from patients with unexplained illness, especially in the context of outbreaks and epidemics [70,71]. As mentioned in the above section, application of high throughput NGS methods in viral metagenomics can greatly enhance the chances to identify viruses in clinical samples, including viruses that are too divergent from known viruses to be detected by PCR or microarray techniques (reviewed in ref. [20]). An attractive application of metagenomic approaches is the study of influenza, given the constant threat of antigenic drift and shift. Deep sequencing strategies can be used to monitor the emergence of mutations that confer virulence or resistance to antiviral drugs, to detect influenza viruses in clinical samples, and to identify viral quasispecies [22,31,32]. In addition, deep sequencing of clinical samples allows to identify and characterize not only novel pathogens but also the microbiota and host response to infection [32].

The study of the human virome includes also the description of viral communities—including bacteriophages—in human body and their relationship with health and disease. Examples are the characterization of fecal viromes (mainly phages) and their relations with bacterial metagenome [72] and the characterization of the virome in the skin of healthy individuals [28].

### 2.4. Full-Length Viral Genome Sequencing

Like viral metagenomics, sequencing of full-length viral genomes is a difficult task due to the presence of contaminating nucleic acids of the host cell and other agents in viral isolates. In fact, preparation of a simple shotgun sequencing DNA library, the most comprehensive approach, or of a library of cDNA synthesized from RNA with random priming, results in a huge amount of host specific instead of a comprehensive representation of the viral sequences, even in the presence of a very high viral load [21,31,73]. Very high throughput sequencing techniques, such as SOLiD platform, could be used to obtain sufficient sequence coverage [74], but the length of reads might be too short to allow *de novo* assembly of viral genomes and methods that provide longer reads, like 454 and Illumina technology, might be preferable [31,32]. Several techniques have been used to enrich virions or viral nucleic acids from cell culture or from host tissue and fluids before extracting the genomic DNA/RNA, in order to limit the contamination from host nucleic acids. One of these methods is ultracentrifugation, but this procedure may be very time-consuming and laborious with uncertain outcome [75]. Other methods are based on enrichment of viral nucleic acids by using capture probes or PCR amplification targeting conserved genome segments [76,77] or, vice versa, by depletion of host nucleic acids by probing total RNA with labeled host nucleic acid [78]. Other approaches could be enrichment of dsRNA virus genomes [79] or circular dsDNA viral genomes by RCA [28,29].

### 2.5. Investigation of Viral Genome Variability and Characterization of Viral Quasispecies

High mutation rates inherent to replication of RNA viruses create a wide variety of mutants that are present in virus populations, which are often referred to as quasispecies [80]. The diffuse, “cloud-like” nature of viral populations allows them to rapidly adapt to changing replicative environments by selecting preexisting variants with better fitness [81,82]. Thus, many important virus properties cannot be explained by a mere consensus sequence, but require knowledge about the microvariants present in viral populations. These sequence variants may be critically relevant to viral evolution and spread, virulence, evasion of the immune response, anti-viral drug resistance, and vaccine development and manufacture.

The use of deep sequencing data for mutation analysis in viral genomes has required the development of computational methods for estimation of the quality of sequences and for error correction, algorithms for sequence alignment and haplotype reconstruction, statistical models to infer the frequencies of the haplotypes in the population, for comparative analysis and for their visualization [83–86].

Among RNA viruses, HIV quasispecies have been extensively investigated because of their relevance for vaccine design and response to antiviral drug therapy [87]. Within infected individuals, HIV is highly heterogeneous owing to rapid turnover rates, high viral load, and a replication mediated by the error-prone reverse transcriptase enzyme that lacks proofreading activity. High variability is also the consequence of recombination, which can shuttle mutations between viral genomes and lead to major antigenic shifts or alterations in virulence [88]. An example of application of NGS for analysis of HIV quasispecies is the use of massive parallel 454 pyrosequencing with the shotgun approach to characterize the full length genome of an HIV-1 BF recombinant and its quasispecies heterogeneity in a patient who died from multiorgan failure during seroconversion [89]. Another fascinating application of deep sequencing in HIV research is the use of the 454 pyrosequencing methods to analyze the variable regions of heavy and light chains of neutralizing antibodies against HIV in the blood obtained from HIV-1-infected individuals, in order to understand how broadly neutralizing antibodies develop [90]. But the most relevant application of NGS in HIV diagnostics is the detection of anti-viral drug resistant minor variants, which will be discussed in the next section.

Analysis of full-length viral genome and quasispecies was also applied to other RNA viruses. Deep sequencing with the Illumina platform on total RNAs extracted from the lung of a patient who died of viral pneumonia due to pandemic 2009 influenza A virus (A/H1N1/2009) revealed nucleotide heterogeneity on hemagglutinin as quasispecies, leading to amino acid changes on antigenic sites which could be relevant for antigenic drift [31].

Mutations of human rhinovirus (HRV) genome were explored in a lung transplant recipient infected with the same HRV strain for more than two years [91]. Analysis of complete HRV genome sequences by both classical and Illumina ultra-deep sequencing of samples collected at different time points in the upper and lower respiratory tracts showed that HRV populations in the upper and lower respiratory tract were phylogenetically indistinguishable over the course of infection, likely because of constant viral population mixing. Nevertheless, signatures of putative adaptation to lower airway conditions appeared after several months of infection, with the occurrence of specific changes in the 5’UTR polypyrimidine tract and the VP2 immunogenic site 2 of HRV genome, which might have been relevant for viral growth at lower airway conditions [91].

Populations of DNA viruses are considered less complex and variable when compared to RNA viruses. However, data from deep sequencing of DNA virus genomes have revealed that complex mixtures of viral genotypes may be present in infected subjects and that positive selection could have contributed to the divergence of different strains. This is the case of human cytomegalovirus (HCMV), which establishes lifelong latent infections in humans and may reactivate and cause severe life-threatening disease in immunocompromised patients. High intra-host variability of HCMV genome was demonstrated in lung transplant recipients by deep sequencing of the amplicons of three variable HCMV genes [92] and in neonates with congenital HCMV infection by deep sequencing of long range, overlapping amplicons covering the entire HCMV genome [93]. Since PCR amplification and sequencing can introduce errors in their own, which could be misinterpreted as mutation or polymorphisms, deep sequencing studies have to develop protocols and algorithms to estimate experimental error and to filter false positive results. In the studies reported here on HCMV genome variability, experimental error rate was estimated by using arbitrary criteria [92] or an algorithm based on experimental data obtained from deep sequencing analysis of a control HCMV genome cloned in a BAC vector [93].

Deep sequencing showed also variability of herpes simplex virus 1 (HSV-1) genome and allowed to demonstrate virulence genes. Using Illumina high-throughput sequencing, genome sequences of both a laboratory strain (F) and a low-passage clinical isolate (H129) were obtained and compared with the available genome sequence of a more virulent isolate of HSV-1 (strain 17) [94]. The HSV-1 H129 strain, isolated from the brain of an encephalitic patient, is the only virus known to transit neural circuits exclusively in an anterograde direction [95]. Whole genome sequencing demonstrated many protein-coding variations between strains F and H129 and the genome reference strain 17 and some genes were proposed to be responsible of the anterograde mutant phenotype of strain H129, including the neurovirulence protein ICP34.5, while a frameshift mutation in the UL13 kinase could account for decreased neurovirulence of strain F [94].

### 2.6. Monitoring Antiviral Drug Resistance

Deep sequencing by NGS techniques is being increasingly used in the clinical practice to detect low abundance drug resistant HIV variants and, with the recent availability of new drugs active against hepatitis C virus (HCV), also for the detection of HCV minor variants.

Conventional direct sequencing of RT-PCR products (referred to as “population sequencing”) is the gold standard in HIV resistance testing and is used to detect drug-resistance mutations in the molecular targets of HIV-1 therapy, *i.e.*, reverse transcriptase, protease, integrase, and V3 loop of the HIV *env* gene. A major limitation of direct PCR sequencing, however, is its inability to detect drug-resistant variants present in less than 20–25% of the heterogeneous virus population existing in a patient’s plasma sample [96]. Several studies have shown that minor drug-resistant variants that are not detected by population-based sequencing are clinically relevant in that they are often responsible for the virological failure of a new antiretroviral treatment regimen [97–99].

Clonal sequencing of RT-PCR products by 454 pyrosequencing offers the advantage of high sensitivity for minor variants and a relatively long sequence length that facilitates the characterization of the linkage amongst resistance mutations and avoids the risk to miss mutations due to sequence variation around the site under investigation. The application of 454 sequencing-based resistance testing in clinical setting, however, requires careful consideration of potential technical errors that can be introduced in the experimental protocol and in data analysis in order to discriminate between experimentally introduced errors and true variants [87,100,101]. Data analysis issues are discussed in Section 2.9.

Several studies that employed 454 pyrosequencing for deep analysis of mutations in HIV protease and reverse transcriptase genes demonstrated the accuracy of this technique in detecting all drug-resistance mutations identified by population sequencing, and the ability to detect low-frequency mutations undetectable by population sequencing [100,102,103]. In addition, several studies demonstrated that drug-resistance mutations detected by 454 had a significant impact on virological failure [103–107] while others did not find a strong association of low-frequency mutations with clinical responses [108,109]. Deep sequencing using the 454 platform has been also applied to investigate drug-resistance mutations against the more recently approved integrase inhibitors and CCR5 antagonists.

Drug-resistance mutations to integrase inhibitors occur in the integrase gene. These mutations were detected by deep sequencing at very low levels if at all prior to initiating therapy [110] and could be selected by previous drug pressure [111]. Resistance to CCR5 antagonists, like maraviroc, occur by outgrowth of CXCR4-tropic HIV variants, *i.e.*, viruses that use the CXCR4 coreceptor [112] or via mutations in the viral envelope protein [113–116]. Coreceptor usage can be screened using phenotypic coreceptor tropism assays, based on recombinant virus technology, or genotypic tests, based on sequencing of the V3 loop of HIV *env* gene [117]. Phenotypic assays have good sensitivity and specificity, but they are time consuming, expensive, and require special laboratory facilities; thus they are not convenient as diagnostic tests in clinical practice. Genotyping methods based on population sequencing represent a more feasible alternative, but their sensitivity for the detection of minority variants is lower than phenotypic assay (about 10–20%) and this represents a problem, since the proportion of CXCR4-tropic HIV variants before initiation of therapy is generally very low. In addition, the algorithms used for interpretation of sequencing results may underestimate the impact of some mutations in viral tropism [118]. Deep sequencing by using 454 has been used in several studies [119–123], including large clinical trials, to determine viral tropism and has been demonstrated to be comparable in sensitivity and specificity with phenotypic assays in detecting CXCR4-using variants. According to data reported to date, the clinical threshold for detection of CXCR4-tropic variants might range between 2–10% [118]. With this threshold, 454 pyrosequencing at ≥1% sensitivity for minority variants can represent a valuable diagnostic tool for viral tropism testing. In addition, deep sequencing of relatively long reads allows defining the contribution of multiple mutations in a single viral genome. This information could improve the performance of interpretation algorithms as compared with population sequencing.

Deep sequencing based on the 454 technology has been also applied for the detection of nucleoside and nucleotide reverse-transcriptase inhibitor resistance in HBV. The NGS method was more sensitive for the detection of rare HBV drug resistance mutations than conventional methods based on population sequencing or reverse hybridization [124,125]. In addition, deep sequencing allowed to identify G-to-A hypermutation mediated by the apolipoprotein B mRNA editing enzyme, which was estimated to be present in 0.6% of reverse-transcriptase genes [124].

Finally, with the availability of new drugs targeting HCV protease and polymerase, the experience of drug-resistance mutation and quasispecies analysis achieved with HIV is being translated to HCV. Also for HCV, deep sequencing technologies seem a promising tool for the study of minority variants present in the HCV quasispecies population at baseline and during antiviral drug pressure, giving new insights into the dynamics of resistance acquisition by HCV [126,127].

### 2.7. Epidemiology of Viral Infections and Viral Evolution

High throughput sequencing is being used to investigate the epidemiology of viral infections and viral evolution, addressing issues such as viral superinfection (e.g., HIV superinfection, which occurs when a previously infected individual acquires a new distinct HIV strain) [128], tracing the evolution and spread of viral strains, such as the emergence, evolution and worldwide spread of HIV [88], tracing the transmission of viruses among individuals [129], or modeling the evolution of viruses within the host and the mechanism of immune escape, balanced with replication fitness, such as in the case of HIV and HCV infection [127,130,131].

### 2.8. Quality Control of Live-Attenuated Viral Vaccines

Intrinsic genetic instability of RNA viruses may lead to the accumulation of virulent revertants during manufacture of live viral vaccines, requiring rigorous quality control to ensure vaccine safety. High throughput deep sequencing methods have been proposed as tools for monitoring genetic consistency of live viral vaccines. Deep sequencing was used to analyze lots of oral poliovirus vaccine and the detected neurovirulence mutations were identical to the mutation detected with the standard method based on PCR and restriction enzyme cleavage [132]. Patterns of mutations present at a low level in vaccine preparations were characteristic of seed viruses used for their manufacture and could be used for identification of individual batches [132]. Deep sequencing was also used to examine eight live-attenuated viral vaccines, *i.e.*, trivalent oral poliovirus, rubella, measles, yellow fever, varicella-zoster, multivalent measles/mumps/rubella, and two rotavirus live vaccines [133]. The method allowed identification of, not only mutations and minority variants relative to vaccine strains, but also sequences of adventitious viruses from the producer avian and primate cells. The results were in agreement with those obtained by using a panmicrobial microarray [133].

### 2.9. Data Analysis Issues

An aspect that should not be neglected when dealing with NGS data, is the bioinformatics analysis and issues concerning sequencing output. There are inherent strengths and weaknesses in the different platforms as reported in Table 1. For example, 454 technology is well suited for small *de novo* sequencing projects and amplicon studies, given its read length output that presently reaches the average length of sequences produced with Sanger method. The main issue to be aware of concerns the homopolymer length, due to signal thresholding of the incorporated nucleotides. SOLiD platform is not presently suitable for amplicon studies due to the short read length, but exhibits an extremely high throughput capacity. Illumina has a superior read length and is not affected by homopolymers but, as SOLiD, shows low coverage of AT rich regions [134]. Other platforms present in Table 1 (with the exclusion of the GS Junior, which shares the same features of 454 FLX but has a lower throughput) are still in development and not yet evaluated in their diagnostic potential.

Besides the specific limits of the different platforms, other common issues should be taken into account and carefully considered. The first sources of problems are certainly chimerical sequences, point mutations and insertions/deletions which occur during reverse transcription, PCR amplification or sequencing itself. In addition, PCR amplification bias might impact the relative frequencies of viral variants. The process of “data cleaning” consists of three main steps: sequence filtering, alignment and error correction, for which a panel of methods has been proposed [84,135,136]. Briefly, the filtering phase removes the low-quality sequences from the dataset, while the error correction separates true variants from those due to experimental noise. This step is based on the idea that errors are randomly distributed with low frequency, while sequences with real mutations can be clustered and their abundance quantified. A cluster of reads presenting the same mutations represents a haplotype and the size of the cluster is the haplotype frequency. Global haplotypes are more difficult to be identified, since the reads must be assembled in larger contigs and a unique solution in aligning overlapping reads is not guaranteed. To this respect, the advantage of 454 platform for haplotype reconstruction studies is evident, thanks to its longer reads output.

As concerns the data analysis step, a multitude of software has been developed for very different applications of NGS. Nevertheless, if on the one hand this availability of methods greatly eases the task, on the other hand available algorithms for both genome assembly and amplicon analysis present limitations or drawbacks [137] which require custom made scripting and in-house resolution of bioinformatics problems caused by specific needs [46]. The direct consequence is that data analysis can be no more sustained by the wet-lab researcher alone, but requires the acquisition of computer skills and bioinformatics expertise.

## 3. Conclusions

Next-generation high throughput sequencing technologies have become available in the last few years and are in continuous development and improvement. They have been widely used in many projects, e.g., whole genome sequencing, metagenomics, small RNA discovery and RNA sequencing. Their common feature is the extremely high throughput data generation. As a result, new issues have to be addressed in order to exploit the full potential of these new instruments: firstly, the data analysis step has become very time consuming and requires a competent amount of manpower and expertise in bioinformatics; secondly, adequate computing resources are necessary to handle the data produced.

Diagnostic virology is one of the most successful applications for NGS and exciting results have been achieved in the discovery and characterization of new viruses, detection of unexpected viral pathogens in clinical specimen, ultrasensitive monitoring of antiviral drug resistance, investigation of viral diversity, evolution and spread, and evaluation of the human virome. With the decrease of costs and improvement of turnaround time, these techniques will probably become essential diagnostic tools in clinical routines.

## Figures and Tables

**Table 1:** Features of “next-generation” sequencing (NGS) platforms.

	Maximum Throughput Mb/run	Mean Length (nucleotide)	Error rate *	Applications	Main source of errors
454 FLX	700	~800 (for shotgun experiments) ~400 (for amplicon experiments)	10^−3^–10^−4^	*De novo* genome sequencing and resequencing, target resequencing, genotyping, metagenomics	Intensity cutoff, homopolymers, signal cross-talk interference among neighbors, amplification, mixed beads
Illumina	6,000	~100	10^−2^–10^−3^	Genome resequencing, quantitative transcriptomics, genotyping, metagenomics	Signal interference among neighboring clusters, homopolymers, phasing, nucleotide labeling, amplification, low coverage of AT rich regions
SOLiD	20,000	~50	10^−2^–10^−3^	Genome resequencing, quantitative transcriptomics, genotyping	Signal interference among neighbours, phasing, nucleotide labeling, signal degradation, mixed beads, low coverage of AT rich regions
Helicos	21,000–35,000	~35	10^−2^	Non amplifiable samples, PCR free and unbiased quantitative analyses	Polymerase employed, molecule loss, low intensities
Ion Torrent PGM	1,000	~200	3 × 10^−2^	De novo genome sequencing and resequencing, target resequencing, genotyping, RNA-seq on low-complexity transcriptome, metagenomics	Homopolymers, amplification
GS Junior	~35	~400	10^−3^–10^−4^	Target resequencing (amplicons), genotyping	Intensity cutoff, homopolymers, signal cross-talk interference among neighbors, amplification, mixed beads

* Error rate considering only substitutions and not insertions/deletions.
